# Transmittance properties of one-dimensional metamaterial nanocomposite photonic crystal in GHz range

**DOI:** 10.1038/s41598-022-21455-2

**Published:** 2022-10-31

**Authors:** Aliaa G. Mohamed, Hussein A. Elsayed, Ahmed Mehaney, Arafa H. Aly, Walied Sabra

**Affiliations:** TH-PPM Group, Physics Department, Faculty of Science, Beni-Suef University, Beni-Suef, 62521 Egypt

**Keywords:** Materials science, Optics and photonics, Physics

## Abstract

We have theoretically demonstrated and explored the transmittance characteristics of a one-dimensional binary photonic crystal composed of metamaterial (MM) and nanocomposite (NC) layers. The NC layer was designed from silver nanoparticles (Ag-NPs) in a host material as Yttrium oxide (Y_2_O_3_). Using the transfer matrix approach (TMM), the optical properties of a one-dimensional binary periodic structure having MM and NC layers in the Giga Hertz (GHz) range were examined. The filling fractions of nanoparticles have been explored to see their effect on the effective permittivity of NC materials. Furthermore, the transmittance properties of the suggested structure were investigated at various incident angles for the transverse electric (TE) polarization. In addition to that, different parameters, such as the thickness of the MM layer, the permittivity of the host dielectric material, the filling fraction, and the thickness of the NC layer are also taken into account. We also discussed the effect of these parameters on the width of the photonic bandgap (PBG). With the optimum values of the optical parameters of NC layer, this research could open the way for better photonic crystal circuits, splitters, switches and others.

## Introduction

In the last decade, metamaterials (MMs) have provided completely novel ways for researchers to overcome some disabilities existed by conventional materials^1,2^. The physical properties of metamaterials (MMs) are essentially determined by the internal, specialized structures of MMs, rather than the intrinsic properties of chemical ingredients. MMs are structures that have been designed to have characteristics such as a negative refractive index (Left-handed)^3^ or an epsilon-near-zero (ENZ)^4^. In addition, non-magnetic split-ring resonators^5,6^, short-slab pairs^7^, and cascaded fishnets^8^ are different designs of MM structures that have been applied in different applications. In this regard, MMs are designed in the form of a metallic fork between two metal split-ring resonators^9^. Such strategy led to the introduction of a double negative refractive index in a specific frequency band^10^. Thus, the response of MMs to the incident electromagnetic waves (EMWs) can provide intriguing physical features that cannot be investigated in naturally occurring or chemically manufactured materials^7,8^. This is why MMs, which literally means "materials beyond natural ones," are used to describe such composite structures.

The creation of artificial materials (the so-called MMs) in which the electromagnetic characteristics have been represented by permeability and permittivity, may be regulated is one of many research fields in nanophotonics. Electromagnetic waves incident on a MM are only affected by the materials effective properties, such as magnetic permeability $\mu$ and electric permittivity $\varepsilon$. The index of refraction, n, which is defined as ${n}^{2}={\mu }_{r}{\varepsilon }_{r}$, is used to describe electromagnetic propagation within materials. Two components of the refractive index can be exerted from this equation as: effective electric permittivity,${\varepsilon }_{r}$ and effective magnetic permeability ${\mu }_{r}$. Veselago^11^ discovered two possible refractive index solutions: (i) when ${\varepsilon }_{r}$ and ${\mu }_{r}$ are positive, the refractive index will be greater than zero (*n* > 0), and (ii) when ${\varepsilon }_{r}$ and ${\mu }_{r}$ are negative, the refractive index will *n* < 0. We intend to produce MMs with a negative effective refractive index by overlapping two sets of meta-structures: ${\varepsilon }_{r}<0$ and ${\mu }_{r}<0$ in the same frequency window, which were firstly have been proven experimentally by Smith and colleagues in the microwave domain^12,13^, transformation optics^14^, optical hyperlens^15^, and invisible cloaking^16^. In addition, these artificial structures received a distinct contribution through the microwave technology due to their peculiar electrophysical features. In this context, MMs, microwave components with increased bandwidth, distributed amplifiers; zero-order resonators, enhanced microwave filters, and other technologies can be used to create multiband waveguide arrays and systems^17^. Besides, we will briefly review some application trends in this section of the study.

Material science advancements have enabled technological and application breakthroughs. One of these breakthroughs is the ability to control and manipulate photon propagation using artificial structures^18,19^. Photonic crystals are one of the most studied photonic engineered structures (PCs). Band gap (BG) materials, also known as photonic crystals, are periodic structures whose periodic modulation is primarily dependent on the permittivity of PC constituent materials, resulting in Bragg scattering interference^20–25^. The features of the constituent nanoparticles as well as the multilayer porosity, which are important for sensing, lasing, and switching^26,27^, can be used to modify their photonic band gap^28–30^.

Recently, many researchers have advocated the use of NC materials as a suitable alternative to dispersive media^31–34^. Nanoparticles of dispersive media embedded in a host material of a dielectric medium can be used to create NC materials^31,35,36^. Furthermore, the use of NC materials in PCs may aid in the development of new PBGs^32–36^. It also allows reducing absorption values based on the volume fraction of the dispersive medium and the nanoparticles spherical radius^31,37^. Metal-dielectric NCs have recently been suggested as potentially appealing PC materials with unique plasmon resonance optical characteristics^38^. The inclusion of NCs materials based on metal and dielectric components has a pronounced effect on the characteristic of PC such as the utilization of additional PBGs, in addition to managing the values of absorption of the proposed structures.

NC materials are used in a wide range of applications, including environmental cleanup, energy storage and conservation, creative catalysts, transportation and safety, healthcare and medicine, biotechnology, and agriculture^32–35,39^. Furthermore, metal-dielectric NCs PCs plasmonic characteristics have been widely demonstrated in absorption-based systems^40,41^. The optical properties of PCs, particularly absorption characteristics, can be adjusted as a result of using plasmonic NCs as doping elements in the dielectric layer^42–45^. In this context, the Maxwell–Garnett model is utilized to characterize the effective permittivity of NC materials based on the filling fractions of NPs^46,47^. When the damping value of the spherical Ag–NPs increases with temperature, the dielectric permittivity of NCs is regulated, and thus temperature, filling fraction, and radius of the NPs embedded in the host material can be used to control PBG properties. Because the filling fraction and radius of NPs can be easily optimized, NCs can have a significant role in the design of numerous plasmatic devices. Numerous resonances could be formed at defined values of such parameters due to interaction with incident electromagnetic waves (EMWs)^48^. Based on all of the aforementioned breakthroughs of NCs through the design and fabrication of PC structures, the mainstay of our study is originally dependent on the inclusion of NC layer besides MM in the design of a 1D PCs for GHz applications. In fact, this is the first time to the best of our knowledge in which a NC layer is stacked together with metamaterial to design a 1D PCs through GHz frequencies. The dependence on this type of materials may provide more alternatives towards the tunability of the PBG and the optical properties as well. In particular, the permittivity of NC materials could be tuned by changing some related parameters such as the core radius of NPs, filling fractions and the host medium^34–38^. As a result, when these materials compared with dielectric and superconductor materials, these materials could be an excellent candidate^9,49,53^. Notably, the inclusion of dielectrics with MM may have a limited contribution towards the tunability of PBGs that could lead to a limited role of PCs in many applications through this frequency region^9,53^. Furthermore, the inclusion of superconductors could provide some peculiar properties for the resulting PBGs^49^. However, the special requirements of superconductors near extremely high temperatures appear to be a significant impediment to this idea. Therefore, we believe that NCs can offer a suitable alternate solution for superconductors. Meanwhile, the present theoretical study is based on the well-known characteristic matrix method and the Maxwell–Garnett model foundations. We have investigated the transmittance characteristics of one-dimensional metamaterial nanocomposite PCs. The optical characteristics of the proposed structure were further investigated at various thicknesses of MM, NC layer, effective permittivity of host material, filling fraction, and incident angle.

## Theoretical formulism

**Figure 1:**
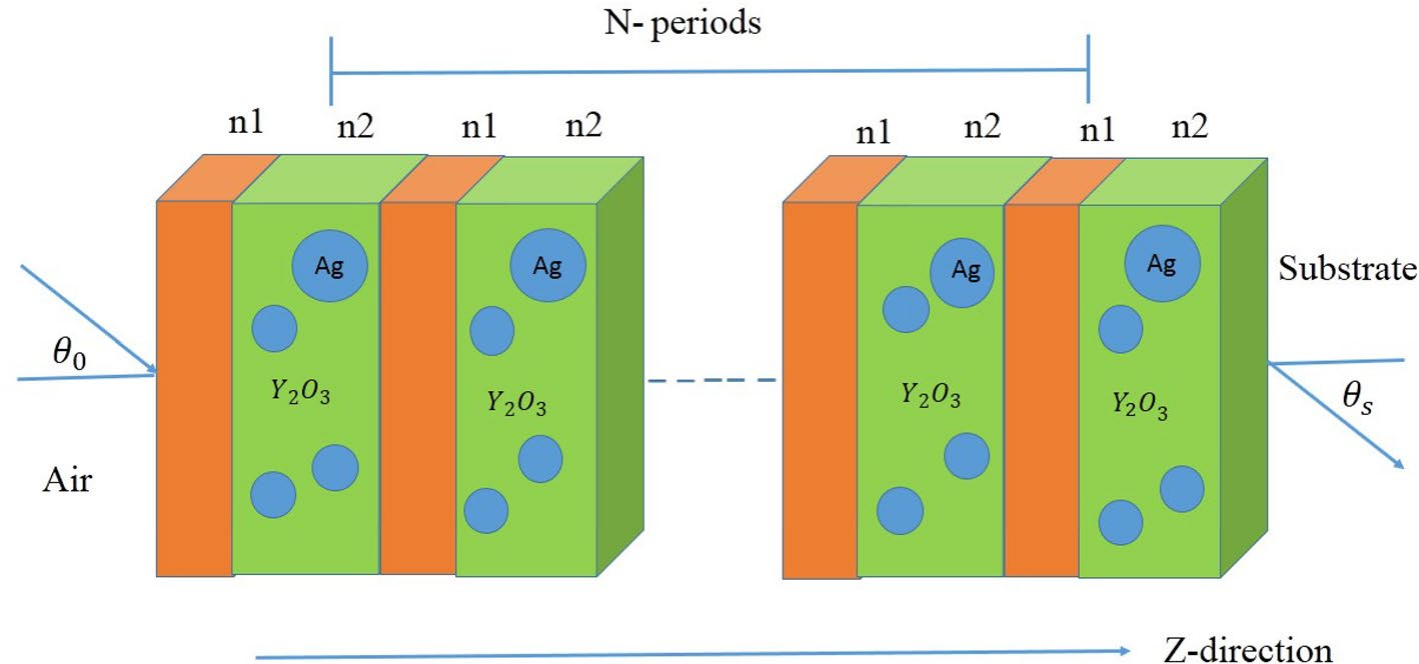
Schematic diagram of the proposed PC structure that configured as ${\left(\mathrm{MM}:\mathrm{Ag}/{\mathrm{Y}}_{2}{\mathrm{O}}_{3}\right)}^{\mathrm{N}}$ with N = 14.

The utilized theoretical framework for our simulation approach is demonstrated in this section. The suggested structure is made up of alternating MM and NC layers, where the first layer is MM (as a layer A) with *d*1 = 30 mm and the second layer is $Ag:{Y}_{2}{O}_{3}$; the NC material (as a layer B) with *d*2 = 10 mm. The values of the radius of the Ag–NPs and filling fraction of the composite are r = 20 nm and $f=5\times {10}^{-1}$, respectively. In this case, we consider the periodicity number of our designed structure (N) = 14. As shown in Fig. 1, the proposed design is sandwiched between a substrate and air as a starting medium that configured as ${\left(MM:Ag/{Y}_{2}{O}_{3}\right)}^{14}$.

In our suggested study, we express the effective electric permittivity ${\varepsilon }_{A}$ and effective magnetic permeability ${\mu }_{A}$ according to the following empirical formula^46^:

1 $$\varepsilon \left(f\right)=1+\frac{{5}^{2}}{{0.9}^{2}-{f}^{2}}+\frac{{10}^{2}}{{11.5}^{2}-{f}^{2}}$$

2 $$\mu \left(f\right)=1+\frac{{3}^{2}}{{0.902}^{2}-{f}^{2}}$$

where *f* is the frequency in the GHz range. Layer A of the proposed construction is a double negative DNG metamaterial operating in the 1 GHz to 3.78 GHz frequency band, with both $\varepsilon$ and $\mu$ being negative. As previously indicated, the MM has unique qualities, such as a negative refractive index in a specific frequency range^49^. This situation is equivalent to $\varepsilon$ and $\mu$ have negative values, and the refractive index is expressed as^50^; ${n}_{A}=-\sqrt{\varepsilon \mu }$.

Here, we show how to calculate the effective permittivity of silver nanoparticles placed in ${Y}_{2}{O}_{3}$ as a host medium. Here, we have utilized Y_2_O_3_ as a host material of the composite because it has a value of permittivity which make the nanocomposite layer more contrasted with the metamaterial layer. In addition, this material is characterized by a very limited value of the extinction coefficient that may lead to reducing the absorption of the incident radiation^51^.

Then, the Plasmon frequency ${\omega }_{p}$ and the decay constant of Ag–NPs are $2\pi \times 2.17\times {10}^{15}\, \mathrm{Hz}$ and $2\pi \times 4.8\times {10}^{12} \, \mathrm{Hz}$, respectively^32–34^. Then, the spherical radius (r) of the Ag–NPs is taken as 20 nm and ${\varepsilon }_{0}=5$^32–34^. The effective permittivity (${\varepsilon }_{eff}$) of silver nanoparticles embedded in ${Y}_{2}{O}_{3}$ is calculated using the Maxwell–Garnett model and is provided by the equation below:

3 $${\varepsilon }_{eff}=\frac{2{\varepsilon }_{b}f\left({\varepsilon }_{m}-{\varepsilon }_{b}\right)+{\varepsilon }_{b}\left({\varepsilon }_{m}+2{\varepsilon }_{b}\right)}{2{\varepsilon }_{b}+{\varepsilon }_{m}+f\left({\varepsilon }_{b}-{\varepsilon }_{m}\right)}$$

where $f$, ${\varepsilon }_{m}$ and ${\varepsilon }_{b}$ are representing the filling fraction (percent) of NPs, Ag–NPs permittivity, and host material permittivity, respectively. The permittivity of Ag–NPs is then calculated using the Drude model^47^ as

4 $${\varepsilon }_{m}={\varepsilon }_{0}-\frac{{\omega }_{p}^{2}}{{\omega }^{2}+i\omega \delta }$$

where ${\varepsilon }_{0}$, ${\omega }_{p}$ and $\delta$ denote the high frequency limit of permittivity, Plasmon frequency, and damping frequency, respectively. At the same time, the radius of NPs(r) and the electron velocity at Fermi energy (v_*f*_) have a crucial role in the nanoparticles damping frequency^34^. In this regard, if the radius of NPs is increased, the damping frequency is decreased according to the following equation:

5 $$\delta \left(r\right)={\delta }_{0}+\frac{{v}_{f}}{r}$$

where, ${\delta }_{0}$ denotes the decay constant. Then, the complex refractive index of the $Ag: {Y}_{2}{O}_{3}$ layer was calculated using the formula ${n}_{B}=\sqrt{{\varepsilon }_{eff}}$.

Then, for a detailed visualization of the response of the incident radiation through our PC structure, the transfer matrix method is the most suitable method to do that. By considering the case of TE mode of polarizations, the components of the electric and magnetic fields through a plane ($x,z$) can be described as^52^:

$${E}_{j}\left(x,z\right)= {A}_{j}\mathrm{exp}\left(i{k}_{j}\left(x,z\right)\right)+{B}_{j}\mathrm{exp}\left(i{k}_{j}\left(x,z\right)\right)={E}_{y+}+{E}_{y-},$$

$${H}_{j}\left(x,z\right)=\frac{-j}{\omega }\frac{\partial E}{\partial \left(x,z\right)}$$

6 $$=\frac{{k}_{j}}{\omega }\left[-{A}_{j}\mathrm{exp}\left(i{k}_{j}\left(x,z\right)\right)+{B}_{j}\mathrm{exp}\left(i{k}_{j}\left(x,z\right)\right)\right]=\frac{{k}_{j}}{\omega }\left[{E}_{y+}-{E}_{y-}\right]={\Omega }_{j}\left[{E}_{y+}-{E}_{y-}\right],$$

$${k}_{j}={k}_{0}{n}_{j}cos{\theta }_{j}.$$

$$\left(\begin{array}{c}{E}_{j}\left(x,z\right)\\ {H}_{j}\left(x,z\right)\end{array}\right)=\left(\begin{array}{cc}1& 1\\ {\Omega }_{j}& -{\Omega }_{j}\end{array}\right)\left(\begin{array}{c}{E}_{y+}\\ {E}_{y-}\end{array}\right),$$

7 $$\left(\begin{array}{c}{E}_{y+}\\ {E}_{y-}\end{array}\right)=\frac{1}{2}\left(\begin{array}{cc}1& {{\Omega }_{j}}^{-1}\\ 1& -{{\Omega }_{j}}^{-1}\end{array}\right)\left(\begin{array}{c}{E}_{j}\left(x,z\right)\\ {H}_{j}\left(x,z\right)\end{array}\right)$$

By considering a specified layer of the whole structure with a thickness ${d}_{j}$, the components of the electric and magnetic fields at the top and bottom surfaces of this layer are described as,

8 $$\left(\begin{array}{c}{E}_{y1+}\\ {E}_{y1-}\end{array}\right)=\left(\begin{array}{cc}\mathrm{exp}\left(i{k}_{j}{d}_{j}\right)& 0\\ 0& \mathrm{exp}\left(-i{k}_{j}{d}_{j}\right)\end{array}\right)\left(\begin{array}{c}{E}_{y2+}\\ {E}_{y2-}\end{array}\right)$$

Therefore, from Eqs. (7) and (8), we have:

$$\left(\begin{array}{c}{E}_{1}\left(x,z\right)\\ {H}_{1}\left(x,z\right)\end{array}\right)=\left(\begin{array}{cc}1& 1\\ {\Omega }_{j}& -{\Omega }_{j}\end{array}\right)\left(\begin{array}{c}{E}_{y1+}\\ {E}_{y1-}\end{array}\right)$$

 = $$\left(\begin{array}{cc}1& 1\\ {\Omega }_{j}& -{\Omega }_{j}\end{array}\right)\left(\begin{array}{cc}\mathrm{exp}\left(i{k}_{j}{d}_{j}\right)& 0\\ 0& \mathrm{exp}\left(-i{k}_{j}{d}_{j}\right)\end{array}\right)\left(\begin{array}{c}{E}_{y2+}\\ {E}_{y2-}\end{array}\right)$$

$$=\frac{1}{2}\left(\begin{array}{cc}1& 1\\ {\Omega }_{j}& -{\Omega }_{j}\end{array}\right)\left(\begin{array}{cc}\mathrm{exp}\left(i{k}_{j}{d}_{j}\right)& 0\\ 0& \mathrm{exp}\left(-i{k}_{j}{d}_{j}\right)\end{array}\right)\left(\begin{array}{cc}1& {{\Omega }_{j}}^{-1}\\ 1& -{{\Omega }_{j}}^{-1}\end{array}\right)\left(\begin{array}{c}{E}_{2}\left(x,z\right)\\ {H}_{2}\left(x,z\right)\end{array}\right)$$

9 $$=\left(\begin{array}{cc}\mathrm{cos}\left({k}_{j}{d}_{j}\right)& -\mathrm{i}{{\Omega }_{j}}^{-1}\mathrm{sin}\left({k}_{j}{d}_{j}\right)\\ -\mathrm{i}{\Omega }_{j}\mathrm{sin}\left({k}_{j}{d}_{j}\right)& \mathrm{cos}\left({k}_{j}{d}_{j}\right)\end{array}\right)\left(\begin{array}{c}{E}_{2}\left(x,z\right)\\ {H}_{2}\left(x,z\right)\end{array}\right)={m}_{j}\left(\begin{array}{c}{E}_{2}\left(x,z\right)\\ {H}_{2}\left(x,z\right)\end{array}\right)$$

where, ${k}_{j}$ describes the wave vector component along ($x$ or $z$) direction, ${\theta }_{j}$ defines the incident angle within layer $j$ and ${m}_{j}$ presents the characteristic matrix that relates the components of the electric and magnetic fields at the top and bottom surfaces of layer $j$. Therefore, the matrix equation that can be used to specify our design based on the matrix described in the previous equation is given as follows,

10 $${M}_{stracture}=\left(\begin{array}{c}{M}_{11} {M}_{12} \\ {M}_{21} {M}_{22} \end{array}\right)={\left({M}_{A}{M}_{B}\right)}^{N}$$

Such that, the characteristic matrices for the MM layer (layer A) and NC layer (layer B) are ${M}_{A}$ and ${M}_{B}$, respectively, where:

11 $${M}_{j}=\left(\begin{array}{c}\mathit{cos}\left({k}_{j}{d}_{j}\right) -\left(i/{\Omega }_{j}\right)\mathit{sin}\left({k}_{j}{d}_{j}\right) \\ -i{\Omega }_{j}\mathit{sin}\left({k}_{j}{d}_{j}\right) \mathit{cos}\left({k}_{j}{d}_{j}\right) \end{array}\right)={\left({M}_{A}{M}_{B}\right)}^{N}$$

$\left({\Omega }_{j}=\sqrt{\frac{{\varepsilon }_{0}}{{\mu }_{0}}}{n}_{j}/\mathrm{cos}{\theta }_{j},j=A,B\right)$. Then, the elements of the matrix in Eq. (10) could define the transmission coefficient as,

12 $$t=\frac{2{p}_{0}}{\left({M}_{11}+{M}_{12}{p}_{s}\right){p}_{0}+\left({M}_{21}+{M}_{22}{p}_{s}\right)},$$

13 $${p}_{0,s}=\sqrt{\frac{{\varepsilon }_{0}}{{\mu }_{0}}}{n}_{0,s}\mathrm{cos}{n}_{0,s},$$

Thus, the transmittance can be given as:

14 $$T=\frac{{p}_{s}}{{p}_{0}}\left|{t}^{2}\right|$$

## Results and discussion

Based on the prior mathematical treatments, the optical features of our design have been introduced in the following sections. Meanwhile, we studied the response of the permeability and permittivity of MM besides the effective permittivity of the NC materials through the frequencies of interest. Then, the transmittance characteristics of the binary 1D-PCs was calculated. In addition, we demonstrated the effect of the host material permittivity, the incident angle of EMW for TE polarization, the thickness of the first and the second layers, and the filling fraction of the NC material on the transmittance characteristic of the proposed PC. Furthermore, we summarize the effect of these parameters on the width of the PBG.

**Figure 2:**
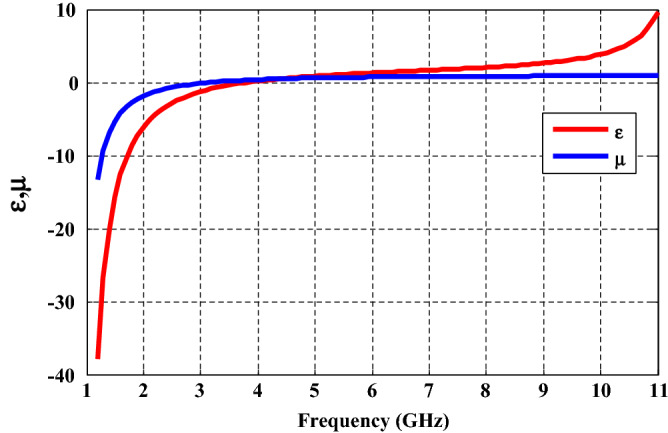
Permittivity and permeability of the MM as a function of frequency at GHz region.

In Fig. 2, we demonstrate the response of the permeability and permittivity of MM through a broad band of GHz frequencies. This figure shows that (layer A) behaves like a DNG metamaterial at the frequency range from 1 to 3.78 GHz, in which both $\varepsilon$ and $\mu$ are simultaneously negative. On the other hand, through the frequency band from 3.5 to 3.78 GHz, the values of $\mu$ are positive while those of $\varepsilon$ are negative, indicating that it is an epsilon-negative (ENG) metamaterial. Both $\varepsilon$ and $\mu$ of the layer get a positive sign at frequency f > 3.78 GHz, and the layer takes positive index material (PIM)^53^. Thus, in our study, we focus on the region of frequencies that give the DNG metamaterial in our structure.

**Figure 3:**
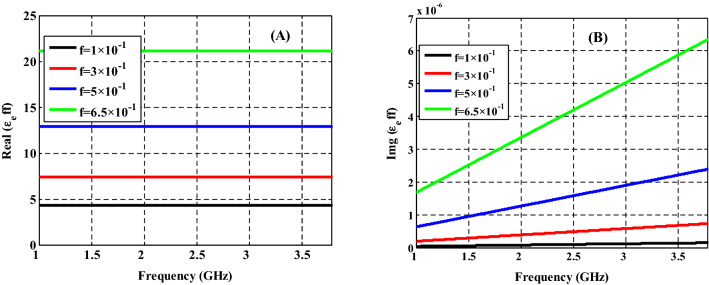
(**A**) Real and (**B**) imaginary parts of the effective permittivity of nanocomposite material at different filling factors of Ag.

The real and imaginary effective permittivity of the NC material are demonstrated in Fig. 3 at the GHz region at constant radius of $Ag$ and different filling fractions of $Ag:{Y}_{2}{O}_{3}$ as $f=1\times {10}^{-1}$, $f=3\times {10}^{-1},f=5\times {10}^{-1}$, and $f=6.5\times {10}^{-1}$. Both the real (Re (${\varepsilon }_{eff}$)) and imaginary (Im (${\varepsilon }_{eff}$)) parts of permittivity are highly influenced by the frequency of incident radiation as well as the filling fraction of nanoparticles.

As illustrated in Fig. 3A, the increase in the filling fraction has a significant effect on the Re (${\varepsilon }_{eff}$). In addition to that, we observe the Re (${\varepsilon }_{eff})$ is almost constant among the frequencies range for each filling fractions. The resulted Re (${\varepsilon }_{eff})$ values are ${\varepsilon }_{eff}=4.29$,${\varepsilon }_{eff}=7.36,\, {\varepsilon }_{eff}=12.88$, and ${\varepsilon }_{eff}=21.16$ at filling fraction values of $f=1\times {10}^{-1}$,$f=3\times {10}^{-1},\,f=5\times {10}^{-1}$, and $f=6.5\times {10}^{-1}$, respectively. Such an effect could be easily clarified with using Eq. (3). This is because the amount of Ag-NPs in the host dielectric substance has been increased with increasing the filling factor. As a result, the dispersion properties of the permittivity of NC material become a significant parameter. Due to the interaction of the incident electromagnetic waves with the surface plasmon of Ag– NPs results in the creation of an asymmetrical mode for both real and imaginary parts which is localized at a certain frequency area^38^. As demonstrated in Fig. 3B, (Im (${\varepsilon }_{eff}$)) becomes independent of the incident radiation frequency ranges where it closes to zero.

**Figure 4:**
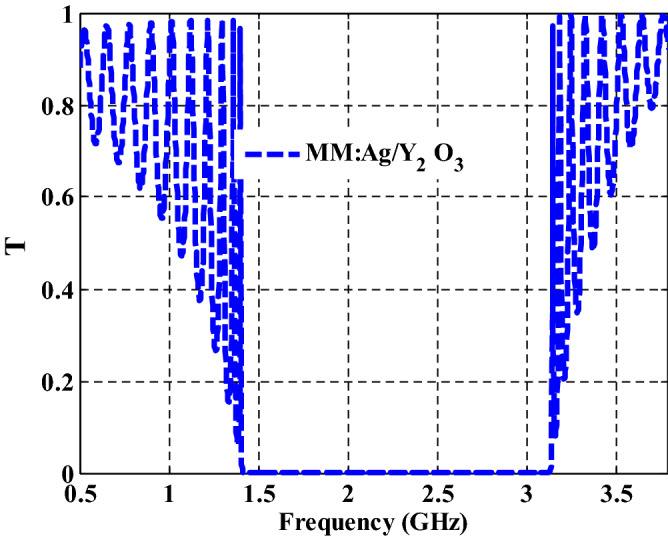
The transmittance characteristics of one-dimensional metamaterial-nanocomposite PCs.

The transmittance of the proposed PC design is investigated in GHz range as shown in Fig. 4. The proposed PC structure is designed based on using both MM and NC materials. From Fig. 4, we observed that there is a Bragg PBG from 1.4 to 3.13 GHz with a broad band width of 1.73 GHz. Here, the PBG is appeared due to the verification of the Bragg condition as illustrated in^17^. In addition to that, a strong refractive index contrast between the two layers, MM and NC, is the cause of forming a wide PBG. Also, from the reasons of appearing this PBG is the constructive interference of the reflected waves at the interface between these different layers^54^. In comparison to the results shown in Fig. 3, the real effective permittivity is changed to 12.88 at filling fraction of $f=5\times {10}^{-1}$ through the GHz range. Moreover, the imaginary effective permittivity at the same frequencies has values near to zero. So that, we can say that the PBG is appeared in this range due to the response of the effective permittivity of NC layer at these frequencies in the GHz range.

**Figure 5:**
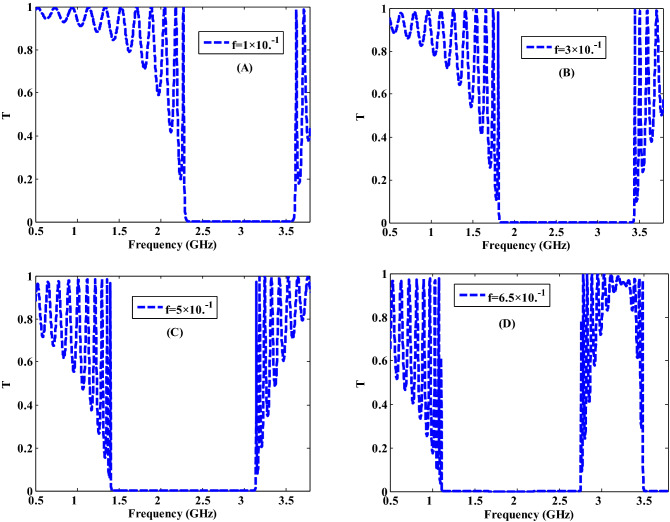
The transmittance characteristics of a one-dimensional metamaterial-nanocomposite PC design at different filling factors of $\mathrm{Ag}:{\mathrm{Y}}_{2}{\mathrm{O}}_{3}$.

Here, we present the effect of the filling fraction on the optical transmittance of our proposed structure as well as the characteristics of the obtained PBGs as follows. Figure 5 depicts the relationship between the width and the locations of the PBGs with changing the filling fraction of Ag-NPs. As shown in Fig. 3, the change in the filling fraction has a significant impact on the effective permittivity of the NC layer, particularly in the real part. This could be owing to the dispersion properties of Ag-NPs, which start to show themselves significantly at high filling fractions. By comparing the results in Fig. 5 with that of Fig. 4, we found that the PBG is slightly shifted to lower frequency values as it is shifted to higher wavelengths with increasing the filling fraction from $1\times {10}^{-1}$ to $3\times {10}^{-1}$, $5\times {10}^{-1}$ and $6.5\times {10}^{-1}$ as presented in Fig. 5A–D, respectively. Because the Ag-NPs increase, the amplitude of the oscillation outside PBG increases as well^55^. Moreover, the width of the PBG increases to 1.63 and 1.73 GHz as shown in Figs. 5B,C, respectively. This response is directly linked to the change in the number of Ag-NPs with changing the filling fraction. This means when the filling fraction increases, the Ag-NPs increase which is leading to the increment in the interaction between the EMW and the suggested structure results in the increase of width of PBG. Furthermore, in Fig. 5D, the PBG width is decreased to 1.66 GHz and a new PBG started to appear at higher frequencies. As shown in Fig. 3, this reaction to the position and width of the PBGs could be attributed to a change in the value of the NC layer effective permittivity to higher values. As a result, a new form of transmittance is expected.

**Figure 6:**
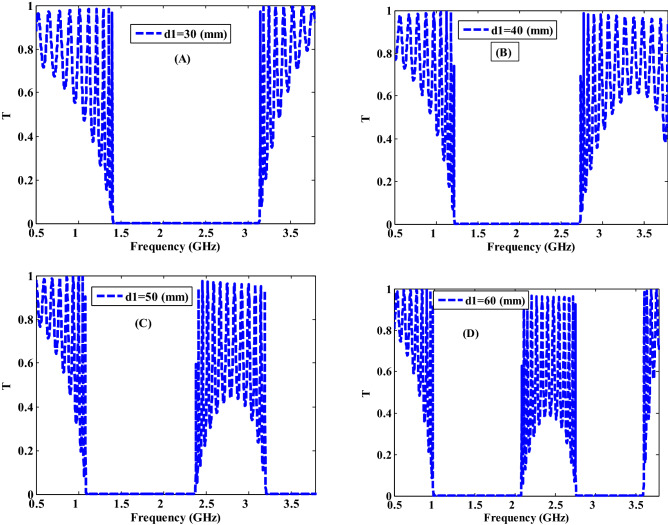
The transmittance characteristics of a one-dimensional metamaterial-nanocomposite PC design at different thickness values of the first layer.

Figure 6 clarifies the proposed PC design transmittance properties at different thicknesses of MM layer as d1 = 30 mm, 40 mm, 50 mm, and 60 mm while the thickness of the second layer (NC) is fixed at d2 = 10 mm at filling fraction $f=5\times {10}^{-1}$. As demonstrated in Fig. 6, an increase in the value of d1 leads to changing the width of the PBGs as a result of the shift to lower frequencies. At d1 = 30 mm only one PBG is appeared in the GHz range with a broad band width (1.73 mm) as shown in Fig. 6A. As the thickness of the MM layer increases to 40 mm and 50 mm, the width of PBG is decreased to 1.53 GHz as depicted in Fig. 6B and 1.29 Fig. 6C, respectively. As a result, the increase in the MM layer thickness leads to a change in the interaction between the EMW and this layer. The width of the PBG is increased in Fig. 6A at d1 = 30 mm then it is decreased in Fig. 6B,C at d1 = 40 mm and at d1 = 50 mm, respectively with appearing a new PBG. Moreover, this PBG is shifted to the lower frequencies because it is shifted to the higher wavelengths with increasing the thickness according to the following equation^56^:

15 $${\varphi }_{i}={2\pi n}_{i}{d}_{i}\mathrm{cos}\left({\theta }_{i}\right)/\lambda$$

where $n$ denotes a refractive index of a layer, $d$ is the thickness of a layer, $\theta$ is the incident angle, $\lambda$ is the wavelength, and $\varphi$ is the phase condition. Furthermore, as the thickness of the MM layer is increased to 60 mm, this PBG is extended to the lower frequencies and the band width is decreased to 1.09 GHz as shown in Fig. 6D. Consequently, the variations in the MM layer thickness result in different responses for the incident EMW path lengths and the transmittance values^56^. In addition, a new PBG is formed at higher frequencies and its width grows progressively to 0.84 GHz as the thickness is increased to d1 = 60 mm, as seen in Fig. 6D. The development of this new PBG can be related to the Bragg PBG shift to the lower frequency areas. Based on the previous results, we can say that the thickness of the MM layer is crucial in determining the width, position, and the number of PBGs.

**Figure. 7:**
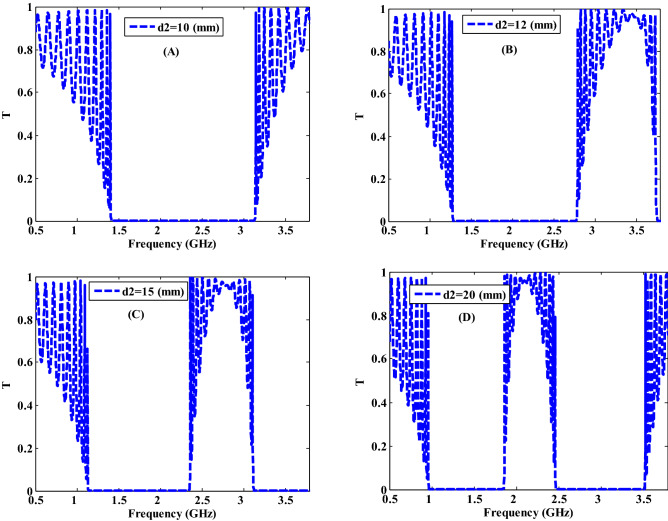
The transmittance characteristics of a one-dimensional metamaterial-nanocomposite PC design at different thickness values of the second layer.

The transmittance properties of the proposed PC at different thicknesses of NC layer has been shown in Fig. 7 at different thicknesses of 10 mm, 12 mm, 15 mm, and 20 mm with fixing the radius of the Ag–NPs, filling fraction of the composite and the thickness of MM at r = 20 nm, $f=5\times {10}^{-1}$ and d1 = 30 mm, respectively. As demonstrated in Fig. 7, the thickness of the NC layer has a significant effect on the properties of the resulted PBGs. For the thickness of the NC layer has the values of d2 = 10, 12, and 15 mm, a pronounced shift of PBG towards the lower frequencies (higher wavelengths) with decreasing the band widths from 1.73 to 1.51 GHz and 1.22 GHz, as shown in Fig. 7A–C, respectively. For further increasing in the thickness of NC layer to 20 mm, the position of this gap is shifted towards the lower frequency values with a width value is decreased to 0.89 GHz as shown in Fig. 7D. Furthermore, at higher frequencies, a new gap with a width of 1.06 GHz begins to form. According to Eq. (15), the increase in the NC layer thickness $d$ will lead to the increase in the wavelength $\lambda$ at constant value of refractive index $n$ and $\mathrm{cos}\left(\theta \right)$. Accordingly, this shift in the PBG is occurred. Also, the width of the PBG decreases and the width of new PBG increases gradually due to increasing the interaction of EMW with NC layer as the increase in the number of Ag-NPs through the NC layer in spite of the value of the filling fraction is constant^57^. As a result, altering the widths, locations, and number of PBGs may be possible by controlling the thicknesses of constituent materials of the proposed PC design.

**Figure 8:**
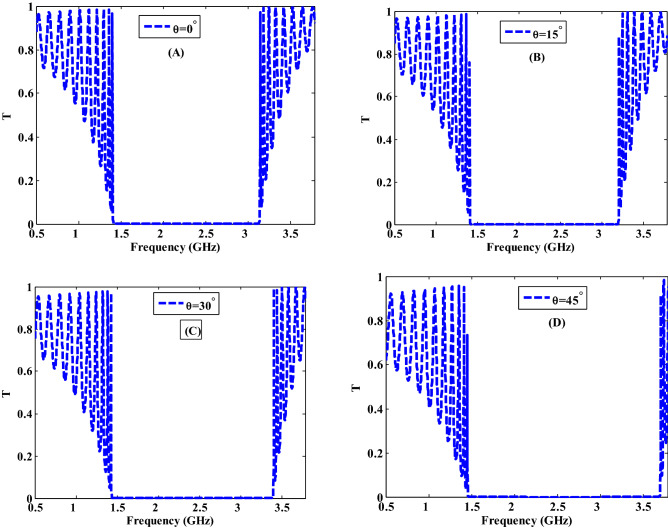
The transmittance characteristics of a one-dimensional metamaterial-nanocomposite PC design at different incident angles.

In Fig. 8, we discussed the influence of the incident angle on the transmittance properties of the suggested PC design for TE polarization. In this study, the incident angle is changed from $\theta ={0}^{^\circ }$ to $\theta ={15}^{^\circ }$ , $\theta ={30}^{^\circ }$ and $\theta ={45}^{^\circ }$. As shown in Fig. 8, the transmittance values are essentially unaffected by increasing the angle of incidence up to 15°. This is especially true for frequencies lower than 1.5 GHz in Fig. 8A,B. As a result, with increasing the incident angles, the width of the PBG has been raised and shifted towards the lower wavelengths (higher frequencies) where it is increased to 1.79 GHz, 1.97 GHz, and 2.24 GHz as shown in Fig. 8B–D, respectively. There are significant decreases in transmittance values for frequencies lower than 1.5 GHz as illustrated in Fig. 8C,D. This can be explained and interpreted according to Eqs. (12) and (13). These equations give the relationship between the transmission and incident angle where we observed that when the angle of incidence increases, the transmission decreases as well.

The shift and the change in the width of the PBG could be attributed to a change in the path length of the incident electromagnetic waves as the angle of incidence changes. The condition of constant phase can be used to describe this phenomenon where it is provided by Eq. (15)^56^. To achieve a phase constant condition, the wavelength $\lambda$ grows as the value $\mathrm{cos}\left(\theta \right)$ increases at constant values of thickness d and refractive index n. As the incident angle is increased, the wavelength must decrease as the value of $\mathrm{cos}\left(\theta \right)$ decreases.

Furthermore, the Bragg-Snell law, which is given by the following equation can be used to explain the behavior of the PBG shift as the incident angle increases^58,59^.

16 $$m\lambda \, = \,2D\sqrt {n_{eff}^{2} - \sin^{2} (\theta )} ,$$

where *m* is the diffracted order,$\lambda$ is the wavelength of light, D is the inter-planar spacing, *n*_*eff*_ is the effective refractive index, and $\theta$ is the incident angle. We can see from this equation that the incident angle is proportional to the incident wavelength. So, with increasing the incident angle, the PBG shifts to the higher frequencies.

**Figure 9:**
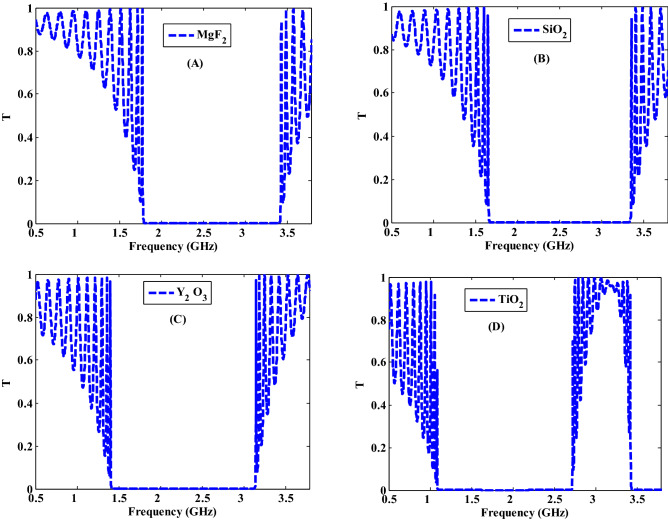
Transmittance characteristic of one-dimensional metamaterial nanocomposite PCs at different host material of $\mathrm{Ag}:{\mathrm{Y}}_{2}{\mathrm{O}}_{3}$.

By following the previous study, we investigated how the permittivity of the host dielectric material can affect the PBGs properties and the transmittance properties as shown in Fig. 9. At filling fraction $f=5\times {10}^{-1}$, we changed the dielectric material from ${\mathrm{MgF}}_{2}$, with permittivity = $1.92$ to ${\mathrm{SiO}}_{2}$ with permittivity = $2.25$, ${\mathrm{Y}}_{2}{\mathrm{O}}_{3}$ and ${\mathrm{TiO}}_{2}$ with permittivity = $3.22$, $5.52$ respectively. Furthermore, the spherical radius of Ag–NPs has been fixed to be $r=20 \mathrm{nm}$. In the case of considering MgF_2_ as a host medium of the NC layer, the transmittance spectrum shows the appearance of the PBG at frequencies from 1.77 to 3.4 GHz with width = 1.63 GHz as shown in Fig. 9A. By replacing ${\mathrm{MgF}}_{2}$ of (${\varepsilon }_{b}=1.92$) with ${\mathrm{SiO}}_{2}$ of (${\varepsilon }_{b}=2.25$), the position of PBG is shifted towards the lower frequencies (higher wavelengths) and its band width increases to 1.68 GHz as shown in Fig. 9B. By using ${\mathrm{Y}}_{2}{\mathrm{O}}_{3}$, the PBG is shifted to lower frequencies and the band width increases to 1.73 GHz as depicted in Fig. 9C. For using ${\mathrm{TiO}}_{2}$ of (${\varepsilon }_{b}=5.52$), the band width decreases to 1.62 GHz and a new PBG is started to appear as illustrated in Fig. 9D. Consequently, the width of the suggested PBG could be regulated based on the host material permittivity. With the substitution of the host dielectric material, the effective permittivity of the NC layer changes (as Eq. 3), resulting in a change in the characteristics of the PBGs. As a result, the contrast in PC refractive indices varies throughout frequencies of interest. As illustrated in Fig. 9, such variance could have a significant impact on the PBG properties. In fact, such response could be similar to that investigated in the cases of angle of incidence, filling fraction and layers thickness variations. However, this parameter could be of a significant interest from real environment view. Notably, the facility of considered a specified host for experimental procedure may collide with the mismatching between the metallic NPs and the host medium. In addition, the change of the host material could lead to the appearance of more than one PBGs as demonstrated in Fig. 9D.

**Figure 10:**
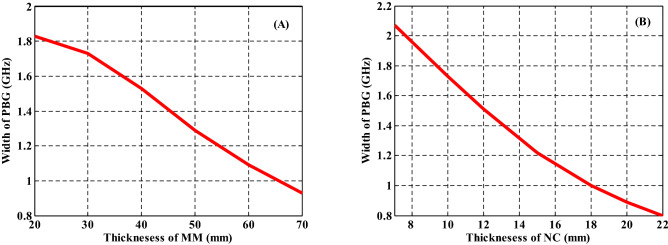
The effect of changing the thicknesses of PC constituent materials on the width of the produced PBG at (**A**) the increase in the thickness of metamaterial layers at constant value of d_2_ = 10 mm and (**B**) the increase in the thickness of NC layers at constant value of d_1_ = 20 mm.

**Figure 11:**
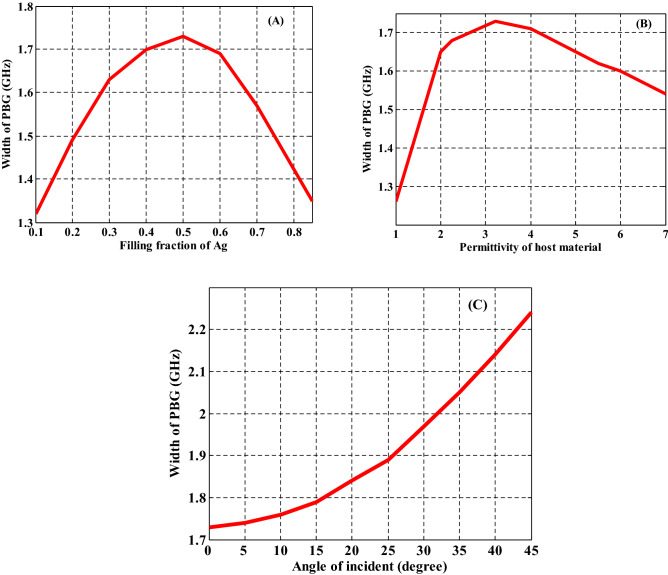
The relation between the width of PBG and different parameters of the proposed PC design such as (**A**) the filling fraction of Ag, (**B**) the permittivity of host material, and (**C**) the incident angle.

Finally, we study the relationship between the width of PBG and the different parameters of the proposed PC design such as the thicknesses of the first and second layers as shown in Fig. 10. The filling fraction, the incident angle, and the dielectric material of NC have been also studied with the width of PBG as shown in Fig. 11. On the other hand, we summarize the previous results in these figures. Figure 10A describes the response of the PBG width regarding the increase in the thickness of MM layers. In fact, this figure summarizes the effect of the thickness of MM layers that was considered in Fig. 6. Here, the increase in the thickness of MM at constant value of nanocomposite layers thickness (d_2_ = 10 mm) leads to a significant decrease in the width of the PBG from 1.83 to 0.93 GHz. Such response is essentially due to the change in the optical path length of the incident electromagnetic waves as demonstrated previously. Then, a similar effect have been investigated in Fig. 10B as the thickness of the nanocomposite layers increases at constant value of metamaterial layers thickness (d_1_ = 20 mm). Figure 10B gives the response of the width of the PBG with increasing the thickness of the NC layer from 10 to 20 mm. We observed that the width of PBG decreases gradually from 2.07 GHz till reaches 0.8 GHz.

Then, we have studied the significance of different parameters on the width of PBG in Fig. 11 such as the effect of different filling fractions of $\mathrm{Ag}:{\mathrm{Y}}_{2}{\mathrm{O}}_{3}$ as shown in Fig. 11A, permittivity of the host material in Fig. 11B, and the significance of incident angle in Fig. 11C. As the filling fraction of $\mathrm{Ag}:{\mathrm{Y}}_{2}{\mathrm{O}}_{3}$ increases from $f=1\times {10}^{-1}$ to $f=8\times {10}^{-1}$, the width of PBG increases to reach 1.73 GHz at $f=5\times {10}^{-1}$, then it is decreased to 1.53 GHz at $f=8\times {10}^{-1}$ as shown in Fig. 11A. This response results due to the changing the number of Ag-NPs with increasing the filling fraction. Figure 11B depicts the width of PBG with increasing the permittivity of the host material from 1 to 7. From this figure, we observed that the PBG width started with 1.26 GHz at ${\varepsilon }_{b}=1$ and increases until it reached 1.73 GHz at permittivity equal ${\varepsilon }_{b}=3.22$. Then, the band width decreases gradually to 1.54 at ${\varepsilon }_{b}=7$. The changing of the contrast in PC refractive indices is the result of the changes in band width of PBG. In Fig. 11C, the incident angle of EMW changes from $\theta ={0}^{^\circ }$ to $\theta ={45}^{^\circ }$. At $\theta ={0}^{^\circ }$, the width of the PBG has the value of 1.73 GHz. Furthermore, by increasing the incident angle its width reached to 2.24 GHz at $\theta ={45}^{^\circ }$. This is due to changing the optical path length with increasing incident angle as showed in Fig. 11C.

## Conclusion

To summarize, by using our theoretical methods, we have demonstrated the optical characteristics of a 1D PC design composed of metamaterial and NC layers. Our theoretical verifications are mostly based on the Maxwell–Garnett model and the transfer matrix approach. The numerical investigation of the dispersion properties of the NC layer may be useful in managing the various characteristics of PBGs. The variation of the design factors such as the MM layer thickness, the background material permittivity, the filling fraction, the incident angle for TE polarization, and the NC layer thickness can also regulate the position and the width of the obtained PBG. PCs based on DNG metamaterials have been the focus of a wide range of fields and applications in recent years, including optics, photonics, optoelectronics, communications, imaging, sensing, and reflectors, polarization filters, and splitters^59^. The proposed structure has the potential to significantly improve the quality and efficiency of the aforementioned fields.

## Data Availability

The datasets used and/or analyzed during the current study are available from the corresponding author on reasonable request.
